# Adjusting for collider bias in genetic association studies using instrumental variable methods

**DOI:** 10.1002/gepi.22455

**Published:** 2022-05-18

**Authors:** Siyang Cai, April Hartley, Osama Mahmoud, Kate Tilling, Frank Dudbridge

**Affiliations:** ^1^ Department of Health Sciences University of Leicester Leicester UK; ^2^ MRC Integrative Epidemiology Unit University of Bristol Bristol UK; ^3^ Department of Mathematical Sciences University of Essex Colchester UK; ^4^ Population Health Sciences, Bristol Medical School University of Bristol Bristol UK

**Keywords:** ascertainment bias, index event bias, Mendelian randomisation, selection bias

## Abstract

Genome‐wide association studies have provided many genetic markers that can be used as instrumental variables to adjust for confounding in epidemiological studies. Recently, the principle has been applied to other forms of bias in observational studies, especially collider bias that arises when conditioning or stratifying on a variable that is associated with the outcome of interest. An important case is in studies of disease progression and survival. Here, we clarify the links between the genetic instrumental variable methods proposed for this problem and the established methods of Mendelian randomisation developed to account for confounding. We highlight the critical importance of weak instrument bias in this context and describe a corrected weighted least‐squares procedure as a simple approach to reduce this bias. We illustrate the range of available methods on two data examples. The first, waist–hip ratio adjusted for body‐mass index, entails statistical adjustment for a quantitative trait. The second, smoking cessation, is a stratified analysis conditional on having initiated smoking. In both cases, we find little effect of collider bias on the primary association results, but this may propagate into more substantial effects on further analyses such as polygenic risk scoring and Mendelian randomisation.

## 1 INTRODUCTION

A major dividend of genome‐wide association studies (GWAS) has been the provision of single‐nucleotide polymorphisms (SNPs) for use as instrumental variables (IVs) to adjust for confounding in epidemiological studies (Hemani, Zheng, et al., 2018). This approach, called Mendelian randomisation (MR), has given rise to a substantial body of methodology as its applications have broadened (Slob & Burgess, 2020). Recently, attention has turned to other forms of bias in observational studies, especially collider bias, which we here regard as a general phenomenon, of which selection, ascertainment and survival biases are well‐known examples (Munafo et al., 2018). Collider bias can occur when the analysis conditions upon a variable that is caused by two or more other variables, whose association is then distorted by the conditioning. Instances of collider bias that have been discussed in genetic epidemiology include secondary analyses in case/control studies (Lin & Zeng, 2009; Monsees et al., 2009), conditioning on heritable covariates (Aschard et al., 2015), case‐only studies of disease progression (Dudbridge et al., 2019), nonrepresentative study cohorts (Yaghootkar et al., 2017) and survival until case recruitment (Anderson et al., 2011; Schooling et al., 2020).

When explanatory variables can model the conditioning process, inverse probability weighting (Monsees et al., 2009) or likelihood approaches (Lin & Zeng, 2009) can be used to adjust for collider bias. When such variables are not available, methods using genetic IVs have been proposed. Zhu et al. (2018) developed a method to adjust for heritable covariates in GWAS, subtracting the covariate‐mediated effect from the total SNP effect on an outcome, where the former is estimated using MR. Dudbridge et al. (2019) and Mahmoud et al. (2022) have proposed regression‐based adjustments with assumptions analogous to those of MR. Pirastu et al. (2021) noted that the Heckman selection model (Heckman, 1979), well known in econometrics, could be applied with genetic IVs, although some practical challenges are present.

Our aim in this paper is to clarify the relationships between the genetic IV methods proposed thus far and to make explicit the links with MR. In so doing, we put the considerable array of MR methodology at our disposal for dealing with collider bias in association studies. We focus on GWAS conditioning on a covariate, but note that some methods may be applied to other settings. We elucidate the relevant assumptions and note where they have contrasting implications from MR studies. We illustrate these aspects with some data examples.

## 2 METHODS

We focus on two situations that have motivated recent development in genetic epidemiology. In the first, the association of an SNP G with an outcome Y is statistically adjusted for a covariate X that may mediate this association. This is often done when seeking effects of G acting through pathways other than those affecting X, for example, GWAS of waist–hip ratio (WHR) adjusting for body mass index (BMI) (Pulit et al., 2019). In the second situation, the effect of G is estimated within a stratum of X, which may reflect selection into the study (Yaghootkar et al., 2017) or the presence of disease, such as in GWAS of progression within cases (Lee et al., 2017).

**Figure 1:**
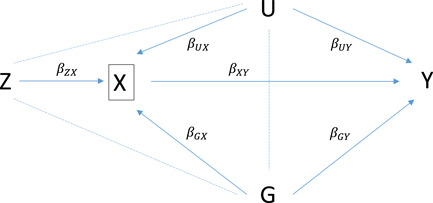
Directed acyclic graph showing the assumed causal structure between a single‐nucleotide polymorphism of interest G, instrumental variable Z, mediating covariate X and outcome Y, with confounder U. Parameters associated with each pairwise association are shown next to the corresponding edges. Conditioning on X is represented by the box and induces moral edges connecting G, Z and U, shown by dashed lines, creating additional paths to Y.

Figure 1 shows a directed acyclic graph describing the causal structure in both situations. Viewed in terms of mediation analysis (Richiardi et al., 2013), the total effect of G on Y comprises its direct effect $\beta_{\mathrm{GY}}$ and its indirect effect through X, a function of $\beta_{\mathrm{GX}}$ and $\beta_{\mathrm{XY}}$. Our interest is in the direct effect, which may be defined as the controlled direct effect or the natural direct effect (VanderWeele, 2013). Both definitions compare the outcomes Y under different (possibly counterfactual) values of G with X held fixed; the controlled direct effect fixes X to a particular value of interest, and the natural direct effect fixes X to its natural value under a reference value of G. When seeking genetic effects through pathways other than through X, the natural direct effect is of interest, whereas effects estimated within strata of X are controlled direct effects. In a study of disease progression within cases, the controlled direct effect is defined for each individual in the population, as the effect on progression if (possibly counter to fact) the individual was to experience the disease.

If there is no interaction between G and X in their effect on Y, the controlled direct and natural direct effects are equivalent (VanderWeele, 2016) and can be estimated by standard regression procedures controlling for or stratifying on X. However, if there are unmeasured confounders U, then this would lead to bias, because conditioning on X would open pathways between Z, G and U (Figure 1).

For simplicity, we assume that the gene G, IV Z and confounder U are univariate, but the methods can be extended to multivariate cases. In particular, multiple SNPs can be used as IVs, and the confounders can include polygenic effects, giving rise to the situations discussed by Aschard et al. (2015). To fix ideas, we will understand Z to be a SNP (as in MR), but any suitable variable could be used as an IV, and indeed a nongenetic variable could potentially be analysed as G in Figure 1.

### 2.1 Conditioning on a covariate: multitrait conditional and joint analysis (mtCOJO)

As part of their mtCOJO, Zhu et al. (2018) developed an approach to condition SNP associations on an intermediate covariate (X in Figure 1). Their aim was to perform MR of pathways not mediated by such variables, but the method has since been applied explicitly in GWAS to identify disease‐specific associations (Byrne et al., 2021).

Simplified to scalar variables with zero mean, mtCOJO assumes linear models relating the variables in Figure 1:

$$X=Z\beta_{\mathrm{ZX}}+G\beta_{\mathrm{GX}}+U\beta_{\mathrm{UX}}+\varepsilon_{X},$$

$$Y=X\beta_{\mathrm{XY}}+G\beta_{\mathrm{GY}}+U\beta_{\mathrm{UY}}+\varepsilon_{Y},$$

(1) $$=Z\beta_{\mathrm{ZX}}\beta_{\mathrm{XY}}+G\left(\beta_{\mathrm{GX}}\beta_{\mathrm{XY}}+\beta_{\mathrm{GY}}\right)+U\left(\beta_{\mathrm{UX}}\beta_{\mathrm{XY}}+\beta_{\mathrm{UY}}\right)+(\varepsilon_{X}\beta_{\mathrm{XY}}+\varepsilon_{Y}).$$

The direct effect of interest is $\beta_{\mathrm{GY}}=\beta_{\mathrm{GY}}^{T}-\beta_{\mathrm{GX}}\beta_{\mathrm{XY}}$, where $\beta_{\mathrm{GY}}^{T}$ is the total effect of G on Y, the terms in Z and U being absorbed into the residual error owing to the collapsibility of the linear model. Subtracted from the total effect is $\beta_{\mathrm{GX}}\beta_{\mathrm{XY}}$, which is the indirect effect of G on Y via X. This requires an estimate of the causal effect $\beta_{\mathrm{XY}}$, which is obtained by MR of X on Y, using Z as the IV. In principle, any MR method could be used to estimate $\beta_{\mathrm{XY}}$, so many SNPs may be used for Z and need not be valid IVs as long as the assumptions for the particular method are met.

The direct effect is obtained from the marginal effects of G and Z on both X and Y. This approach is useful when those marginal effects are available, as is often the case with summary GWAS data. Where only the collider‐biased conditional effects are provided, mtCOJO cannot be used to adjust them toward unbiased effects. In particular, this approach cannot be used to correct within‐stratum effects, as in case‐only studies of prognosis.

### 2.2 Instrument effect regression

Dudbridge et al. (2019) developed an approach based on the same linear model framework as mtCOJO (Equation 1), but which starts from the collider‐biased conditional effects and adjusts them toward the direct effects. Specifically, they showed that the magnitude of collider bias is approximately linear in the effect of G on X:

(2) $$\beta_{\mathrm{GY}}^{C}=\beta_{\mathrm{GY}}+b\beta_{\mathrm{GX}},$$

where $\beta_{\mathrm{GY}}^{C}$ is the conditional effect of G on Y given X. By conditioning on X, we open pathways between G and U (Figure 1), so that the conditional effect is the direct effect of interest plus the bias term. With knowledge of b, the bias‐corrected effect is simply $\beta_{\mathrm{GY}}=\beta_{\mathrm{GY}}^{C}-b\beta_{\mathrm{GX}}$. If Z is a valid IV for the effect of X on Y, then $b=\frac{\beta_{\mathrm{ZY}}^{C}}{\beta_{\mathrm{ZX}}}$ and the problem is analogous to MR in that we estimate a linear relationship between instrument effects on the outcome (here conditional on covariate) and instrument effects on the covariate.

Dudbridge et al. did not assume that any SNPs are valid IVs, and estimated b by a genome‐wide regression of $\beta_{\mathrm{GY}}^{C}$ on $\beta_{\mathrm{GX}}$, including the intercept. This is logically equivalent to MR‐Egger (Bowden et al., 2015); as this is now rather distant from the original formulation (Egger et al., 1997), we will call this procedure *instrument effect regression*. Similar to the InSIDE assumption of MR‐Egger, we assume that the direct effects $\beta_{\mathrm{GY}}$ are independent of (more precisely, uncorrelated with) the effects on covariate $\beta_{\mathrm{GX}}$. As discussed below, this assumption applies to the signed effect sizes, which reflect the allelic coding. We will therefore use the acronym InCLUDE (instrument coefficient linearly uncorrelated with direct effect), as opposed to the instrument strength of InSIDE, which does not recognise the dependence on allele coding.

The InCLUDE assumption may be disputed. GWAS have been performed predominantly on the risk of disease, with the premise that associated SNPs indicate targets for treatment, thus assuming some genetic correlation between incidence and progression. The motivation of Dudbridge et al. was to use as many SNPs as possible in estimating the slope b, so that its sampling variance has little effect on the power of testing $\beta_{\mathrm{GY}}$. They showed that under a positive correlation between direct genetic effects on incidence and progression, the increase in Type‐1 error remains less than for an unadjusted analysis, with a similar level of power.

### 2.3 Slope‐hunter

In contrast, Mahmoud et al. (2022) set out to identify a set of valid IVs from a genome‐wide set of SNPs. Instrument effect regression is then performed on those SNPs only and the estimated slope b is then used to adjust the conditional effects as above.

Identification of the valid IVs follows an algorithmic approach called *Slope‐hunter*. SNPs that affect X are first identified by *p * value thresholding. Model‐based clustering is then used to fit a bivariate normal mixture model to the conditional effects on outcome $\beta_{\mathrm{GY}}^{C}$ and the effects on the covariate $\beta_{\mathrm{GX}}$. One component of this model assumes a proportional relationship between $\beta_{\mathrm{GY}}^{C}$ and $\beta_{\mathrm{GX}}$ and is assumed to identify the valid IVs.

The key assumption of Slope‐hunter is that the model‐based clustering algorithm correctly identifies the valid IVs, and this will tend to be the case when the largest number of similar ratios $\frac{\beta_{\mathrm{GY}}^{C}}{\beta_{\mathrm{GX}}}$ comes from the valid IVs. This resembles the zero modal pleiotropy assumption of the mode‐based estimator in MR (Hartwig et al., 2017); in this context, Mahmoud et al. have called the assumption ZEMRA (zero modal residual assumption) to reflect the emphasis on the residuals rather than the slope of the regression of $\beta_{\mathrm{GY}}^{C}$ on $\beta_{\mathrm{GX}}$.

Under similar simulations to those of Dudbridge et al. (2019), Slope‐hunter had correct Type‐1 error and increased power over instrument effect regression even under genetic correlation. The exception was under a strong negative correlation with the valid IVs explaining no more variation in X than the invalid IVs. In this case, both methods had increased Type‐1 error and reduced power compared with the unadjusted analysis. In other simulated scenarios, in which the confounding was strong and many SNPs were associated with the outcome, Slope‐hunter had the better performance.

### 2.4 MR methods

The above approaches have been proposed specifically for situations entailing collider bias. However, any MR method could be used to estimate the slope in Equation (2) using summary estimates of $\beta_{\mathrm{ZX}}$ and $\beta_{\mathrm{ZY}}^{C}$ with equivalent assumptions on instrument validity. Instrument effect regression is logically equivalent to MR‐Egger, with the InCLUDE assumption and in principle allowing unbalanced direct effects, $E(\beta_{\mathrm{GY}})\neq 0$. Slope‐hunter has the same assumption as mode‐based MR and is similar to MR‐Mix (Qi & Chatterjee, 2019) in that a two‐component mixture is fitted to the SNP effects. Whereas Slope‐hunter fits a bivariate model to the pair of effects ${(\beta }_{\mathrm{GX}},\beta_{\mathrm{GY}}^{C})$, MR‐Mix fits a univariate model to the residual $\beta_{\mathrm{GY}}^{C}-b\beta_{\mathrm{GX}}$. Median‐based estimators (Bowden, Davey Smith, et al., 2016) may also be entertained. In practice, the IV assumptions cannot be verified, and a range of analyses should be performed aiming to observe consistent results.

There are important points of contrast with MR. First, sample overlap does not affect instrument effect regression or Slope‐hunter. This is because any covariance between sample estimates ${\hat{\beta }}_{\mathrm{GY}}^{C}$ and ${\hat{\beta }}_{\mathrm{GX}}$ is included in the unmeasured confounder U, but the path through U is explicitly estimated and then removed from the biased estimator ${\hat{\beta }}_{\mathrm{GY}}^{C}$. Therefore, instrument effect regression is analogous to two‐sample MR even when conducted within a single sample, a property demonstrated in simulations (Dudbridge et al., 2019; Mahmoud et al., 2022). mtCOJO, however, which utilises standard MR, is affected by sample overlap in the usual way (Burgess et al., 2016).

In the two‐sample setting, weak instruments act to bias the estimated slope toward the null (Bowden, Del Greco, et al., 2016; Pierce & Burgess, 2013). In MR this leads to conservative inferences, and although the issue is well known, it is currently unusual for applied studies to enact corrections for weak instruments. However, in the collider bias context, the effect is to underestimate the slope and hence underadjust the conditional estimates for unmeasured confounding.

Whereas in MR the direct effects of G on Y are nuisance parameters that are in various ways ostracised from the analysis (Hemani, Bowden, et al., 2018), they are the parameters of interest in the collider bias setting and nonzero values are explicitly sought. If a limited number of SNPs are used as IVs, with strong associations with X, it seems difficult to justify the InCLUDE or ZEMRA assumptions for their direct effects on Y. However, such assumptions may be more plausible among a very large set of SNPs. Precise estimation of the bias correction is desirable to retain power from the unadjusted analysis, especially in exploratory GWAS settings where the discovery of multiple associations is the priority. To that effect, some bias in the slope estimation may be acceptable, in contrast to MR where unbiased estimation and hypothesis testing of the causal effect is favoured.

These considerations point to the use of a large set of SNPs as IVs, which will generally include many weak IVs that could lead to underadjustment for collider bias. Consideration of weak instrument bias is therefore particularly important in the collider bias setting.

### 2.5 Weak instruments

Simulations of GWAS‐scale data suggest that without correction for weak instrument bias, instrument effect regression has Type‐1 error rates similar to those of an unadjusted analysis (Dudbridge et al., 2019). Dudbridge et al. suggested two approaches to correct for weak instruments. The first, related to the Hedges–Olkin estimator of random effects variance in meta‐analysis, is a simple function of the summary effect estimates. The second is an implementation of the SIMEX algorithm, first proposed for this problem by Bowden, Del Greco, et al. (2016), modified to give a more precise correction. Here, we give a more general formulation of the first approach, now extended to a weighted regression.

Replacing parameters by their estimates, rewrite Equation (2) as

(3) $${\hat{\beta }}_{\mathrm{GYi}}^{C}=\alpha +b{\hat{\beta }}_{\mathrm{GXi}}+\varepsilon_{\mathrm{GYi}},$$

where i indexes SNPs, the residual $\varepsilon_{\mathrm{GYi}}=(\beta_{\mathrm{GYi}}-\alpha )+({\hat{\beta }}_{\mathrm{GYi}}^{C}-\beta_{\mathrm{GYi}}^{C})$ includes both the direct effect and sampling error, and $E\left(\varepsilon_{\mathrm{GYi}}\right)=0$. By setting b as a constant, we assume that the confounding is the same for all SNPs, which is approximately true when SNPs have small effects. Note that the sampling variances of ${\hat{\beta }}_{\mathrm{GYi}}^{C}$ and ${\hat{\beta }}_{\mathrm{GXi}}$ may differ across SNPs. To allow for variable precision in the ${\hat{\beta }}_{\mathrm{GYi}}^{C}$, we can estimate b by weighted least squares, specifically

$$\hat{b}=\frac{\sum w_{i}\sum w_{i}{\hat{\beta }}_{\mathrm{GXi}}{\hat{\beta }}_{\mathrm{GYi}}^{C}-\sum w_{i}{\hat{\beta }}_{\mathrm{GXi}}\sum w_{i}{\hat{\beta }}_{\mathrm{GYi}}^{C}}{\sum w_{i}\sum w_{i}{\hat{\beta }}_{\mathrm{GXi}}^{2}-{\left(\sum w_{i}{\hat{\beta }}_{\mathrm{GXi}}\right)}^{2}},$$

where $w_{i}$ is the weight of SNP i, typically the inverse sampling variance of ${\hat{\beta }}_{\mathrm{GYi}}^{C}$ normalised so $\sum w_{i}=1$. Note that the residual variance in Equation (2) is greater than the sampling variance by $\mathrm{var}(\beta_{\mathrm{GY}})$ and so the inverse sampling variance weighting is not the most efficient estimator of b.

To deal with the imprecision in ${\hat{\beta }}_{\mathrm{GXi}}$, write $\hat{b}$ in terms of the true but unknown $\beta_{\mathrm{GXi}}$

$$\hat{b}=\frac{\sum w_{i}\sum w_{i}(\beta_{\mathrm{GXi}}+\varepsilon_{\mathrm{GXi}}){\hat{\beta }}_{\mathrm{GYi}}^{C}-\sum w_{i}{(\beta }_{\mathrm{GXi}}+\varepsilon_{\mathrm{GXi}})\sum w_{i}{\hat{\beta }}_{\mathrm{GYi}}^{C}}{\sum w_{i}\sum w_{i}{{(\beta }_{\mathrm{GXi}}+\varepsilon_{\mathrm{GXi}})}^{2}-{\left(\sum w_{i}{(\beta }_{\mathrm{GXi}}+\varepsilon_{\mathrm{GXi}})\right)}^{2}},$$

$$\approx \frac{\sum w_{i}\sum w_{i}\beta_{\mathrm{GXi}}{\hat{\beta }}_{\mathrm{GYi}}^{C}-\sum w_{i}\beta_{\mathrm{GXi}}\sum w_{i}{\hat{\beta }}_{\mathrm{GYi}}^{C}}{\sum w_{i}\sum w_{i}\beta_{\mathrm{GXi}}^{2}-{\left(\sum w_{i}\beta_{\mathrm{GXi}}\right)}^{2}+\sum w_{i}\sum w_{i}\varepsilon_{\mathrm{GXi}}^{2}},$$

where $\varepsilon_{\mathrm{GXi}}$ is the sampling error in ${\hat{\beta }}_{\mathrm{GXi}}$, assumed to have zero mean. Approximate $\sum w_{i}\varepsilon_{\mathrm{GXi}}^{2}$ in the denominator by $\sum w_{i}\sigma_{\mathrm{GXi}}^{2}$, where $\sigma_{\mathrm{GXi}}$ is the (estimated) standard error of ${\hat{\beta }}_{\mathrm{GXi}}$. Then, compared to the estimate based on the true $\beta_{\mathrm{GXi}}$, the numerator is the same, but the denominator is increased by $\sum w_{i}\sum w_{i}\sigma_{\mathrm{GXi}}^{2}$. We, therefore, subtract that term from the observed denominator and obtain the weak‐instrument corrected slope as

(3) $${\hat{b}}_{\mathrm{cor}}=\frac{\sum w_{i}\sum w_{i}{\hat{\beta }}_{\mathrm{GXi}}{\hat{\beta }}_{\mathrm{GYi}}^{C}-\sum w_{i}{\hat{\beta }}_{\mathrm{GXi}}\sum w_{i}{\hat{\beta }}_{\mathrm{GYi}}^{C}}{\sum w_{i}\sum w_{i}{\hat{\beta }}_{\mathrm{GXi}}^{2}-{\left(\sum w_{i}{\hat{\beta }}_{\mathrm{GXi}}\right)}^{2}-\sum w_{i}\sum w_{i}\sigma_{\mathrm{GXi}}^{2}}.$$

A special case is a zero‐intercept model assuming $E\left(\beta_{\mathrm{GY}}\right)=0$, hence α=0. Then,

(4) $${\hat{b}}_{\mathrm{cor}}=\frac{\sum w_{i}{\hat{\beta }}_{\mathrm{GXi}}{\hat{\beta }}_{\mathrm{GYi}}^{C}}{\sum w_{i}{\hat{\beta }}_{\mathrm{GXi}}^{2}-\sum w_{i}\sigma_{\mathrm{GXi}}^{2}}.\;$$

Because we have not estimated the unknown $\mathrm{var}(\beta_{\mathrm{GY}})$, there is residual heteroscedasticity in Equation (3). To estimate the variance of $\hat{b}$ when using a large number of SNPs, we suggest using a sandwich variance estimator, similarly scaled to correct for weak IVs.

We call this approach corrected weighted least squares (CWLS), and will compare it to MR using the robust adjusted profile score (MR‐RAPS) (Zhao et al., 2020), which has been developed for MR using genome‐wide SNPs. MR‐RAPS uses the zero‐intercept model and defines a likelihood for b while also estimating $\mathrm{var}(\beta_{\mathrm{GY}})$. By accounting for that variance in the estimation of b, MR‐RAPS might be more efficient than CWLS.

### 2.6 Allele coding

Instrument effect regression is sensitive to allele coding in that the estimated slope can change depending on which allele of each SNP is taken as the effect allele (Burgess & Thompson, 2017). These changes arise from a violation of the InCLUDE assumption. Equation (2) shows that changing the effect allele preserves the same linear relationship between $\beta_{\mathrm{GY}}^{C}$ and $\beta_{\mathrm{GX}}$ because the signs of all terms are reversed. Any change in the estimated relationship comes from a change in compliance with the model assumptions. In fitting the model via Equation (3), the intercept may change, but the slope is unchanged as long as the InCLUDE assumption holds.

To standardise the allele coding, a common practice is to take the effect allele as the one with a positive effect on X (Burgess & Thompson, 2017). The InCLUDE assumption is then that the direct effect of the X‐increasing allele on Y is uncorrelated with its effect on X. Although this assumption remains unverifiable, it has the desirable property of expressing the assumption only in terms of allelic effects, as opposed to, say, taking the effect allele as the minor allele. Furthermore, when viewing MR as an analogy to a randomised trial, positive allele coding corresponds to each SNP representing the same direction of treatment effect.

However, in practice, the positive effect alleles are identified from estimates ${\hat{\beta }}_{\mathrm{GXi}}$ and this biases the sampling errors toward positive values. This is not a problem when using strongly associated SNPs as IVs, because this ensures that estimates tend to have the same sign as the true effects. But when weak instruments are used, the adjustments discussed above assume sampling errors with a mean zero and are thus invalid when using positive effect coding. In the Results section, we will demonstrate through simulation that such coding can lead to increased Type‐1 error rates. The exception is when the positive effect alleles are identified from an independent data source, a practice also encouraged to reduce the winner's curse in selecting SNPs as IVs (Zhao et al., 2019), but which is currently not widespread.

When the intercept α is set to 0 in Equation (2), the regression is invariant to allele coding. The zero‐intercept assumption is therefore a pragmatic solution to the allele coding problem when many SNPs are used as IVs, as we argue is desirable in the collider bias setting. More precisely, the assumption is that there is an allele coding under which $E\left(\beta_{\mathrm{GY}}\right)=0$ and the InCLUDE assumption holds. This is the assumption made by MR‐RAPS, and as a simple alternative, we also suggest the CWLS estimator (Equation 4) with a sandwich variance estimate.

In summary, we argue that in the collider bias setting it is desirable to use a large number of SNPs as IVs, potentially leading to substantive weak instrument bias. The bias cannot be properly corrected under an MR‐Egger regression model with positive allele coding, so we, therefore, suggest using only the zero‐intercept model, estimated by MR‐RAPS or through a CWLS formula (Equation 4), or by using Slope‐hunter or polygenic MR methods that estimate the slope using an identified set of valid IVs.

### 2.7 Within‐stratum effects

So far, we have considered bias in the conditional effects $\beta_{\mathrm{GY}}^{C}$ marginalised over the conditioning covariate X. In some situations, the interest is within certain strata of X, a particular case being studies of disease progression in which the outcome Y is only observed among cases. The within‐stratum effects need not equal the conditional effect, and the linear relationships (Equations 1 and 2) exploited by the IV methods above need not hold. Even if the within‐stratum effects are constant across X and equal to the conditional effect, it is not given that they are linear in $\beta_{\mathrm{GX}}$ as in Equation (2), because the distribution of U may vary across X.

The Heckman selection model is an established approach using IVs to adjust for selection on a binary event such as disease incidence (Heckman, 1979). In the first step, a probit regression model is fitted to the selection event

$$\text{Pr}\left(X=1|G,Z\right)=\Phi (\alpha +Z\beta_{\mathrm{ZX}}+G\beta_{\mathrm{GX}}).$$

In the second step, the fitted probability is included, after transformation, as a covariate in the regression

$$E\left(Y|G,Z,X=1\right)=G\beta_{\mathrm{GY}}+\frac{\phi \left(\alpha +Z\beta_{\mathrm{ZX}}+G\beta_{\mathrm{GX}}\right)}{1-\Phi \left(\alpha +Z\beta_{\mathrm{ZX}}+G\beta_{\mathrm{GX}}\right)}\gamma ,$$

where γ is a nuisance parameter to be estimated (for simplicity we continue to assume that G, Z and Y have an unconditional mean of zero). The underlying intuition is an assumption that all individuals have a latent (possibly counterfactual) outcome Y whether or not X=1, which is modelled by linear regression with a normally distributed error. Conditional on X=1, the error becomes truncated normal with its mean estimated from the first‐stage model and then included in the second‐stage model.

In its use of IVs and substitution in Stage 2 of the fitted values from Stage 1, the Heckman correction is reminiscent of the two‐stage least‐squares estimate in MR. Similar to that approach, it requires individual‐level data on G and Z. Moreover, the probit model is rarely used in the analysis of single‐SNP data. For these reasons, the Heckman selection model appears problematical if only summary‐level GWAS data are to hand (Pirastu et al., 2021).

However, when SNP effects on X are assumed small, as is the case for polygenic traits, we may take a first‐order approximation to the coefficient of γ above (called the inverse Mills ratio) and write

$$E\left(Y|G,Z,X=1\right)\approx G\left(\beta_{\mathrm{GY}}+b\beta_{\mathrm{GX}}\right)+Zb_{Z}+\gamma^{*}.$$

The within‐stratum coefficient of G now has the same form as the conditional effect in Equation (2), motivating the use of instrument effect regression, Slope‐hunter or polygenic MR methods to estimate b from summary statistics and adjust the within‐stratum effects toward the direct effects $\beta_{\mathrm{GY}}$. Simulations under logistic models for X have suggested acceptable operating characteristics of this approach (Dudbridge et al., 2019).

In Table 1 we summarise the methods discussed throughout this section.

**Table 1:** Properties of some instrumental variable methods to adjust for collider bias

	Condition on covariate	Selected sample	Main assumptions	Summary statistics	Total *G*–*Y*	Conditional *G*–*Y*	Very weak instruments
mtCOJO	Yes	No	(Default) InCLUDE, zero mean direct effect	Yes	Yes	No	No
CWLS	Yes	Yes	InCLUDE, zero mean direct effect	Yes	No	Yes	Yes
MR‐RAPS	Yes	Yes	InCLUDE, zero mean direct effect	Yes	Yes	Yes	Yes
Slope‐hunter	Yes	Yes	ZEMRA	Yes	No	Yes	No
MR‐Mix	Yes	Yes	ZEMRA	Yes	Yes	Yes	No
Weighted mode	Yes	Yes	ZEMRA	Yes	Yes	Yes	No
Weighted median	Yes	Yes	Weighted majority valid	Yes	Yes	Yes	No
Heckman selection model	No	Yes	Probit model of selection	No	No	No	No

*Note*: The columns indicate (1) whether a method can be applied in GWAS conditioning on a covariate, and (2) in GWAS of selected samples; (3) the main assumptions of each method; (4) whether a method can use summary statistics rather than individual‐level data; (5) whether it requires total or (6) conditional effects of *G* on *Y*; (7) whether it accounts for bias from very weak instruments. mtCOJO, CWLS, and Slope‐hunter are developed specifically for collider bias correction and are described according to their current implementations. MR‐RAPS, MR‐Mix, weighted median, and weighted mode are developed for MR, but can be applied to collider bias correction, either by adjusting the marginal effect of *G* on *Y*, as per mtCOJO, or the conditional effect, as per CWLS and Slope‐hunter.

Abbreviations: CWLS, corrected weighted least squares; GWAS, genome‐wide association studies; MR‐RAPS, Mendelian randomisation using the robust adjusted profile score; mtCOJO, multitrait conditional and joint analysis; ZEMRA, zero modal residual assumption.

### 2.8 Data examples

We illustrate the methods on two data examples, the first conditioning on a covariate and the other a within‐stratum analysis. First, we consider the summary statistics in the GWAS of WHR adjusted for BMI conducted by the GIANT consortium (Pulit et al., 2019). That analysis aimed to find SNPs acting on WHR through pathways other than those affecting BMI: conditioning on BMI would block any causal effect of BMI on WHR, or (more plausibly) attenuate effects acting through shared determinants of the two traits. Those authors discussed the possibility of collider bias in the associations of 346 index SNPs and concluded that any bias was small, based on several lines of evidence: the unadjusted effects on WHR were stronger than those on BMI, suggesting the presence of direct effects; the SNP with the strongest effect on BMI was not associated with WHR after adjustment for BMI; and polygenic score effects on WHR adjusted for BMI were consistent with those on WHR and BMI.

To formally quantify the degree of collider bias, we applied IV methods to 143,000 pruned ($r^{2}\leq 0.1$, 250 SNP window) and well‐imputed ($r^{2}\geq 0.98$) SNPs identified in our previous study (Dudbridge et al., 2019) and present in this data set, and adjusted the estimated effects of the index SNPs on WHR adjusted for BMI. We applied CWLS, MR‐RAPS, and Slope‐hunter to all SNPs, and to those passing *p* value thresholds of $10^{-4}$, $10^{-6}$ and $10^{-8}$ to assess the effects of such thresholding. These were compared to the inverse‐variance weighted (IVW) estimator from standard MR analysis, which can be regarded as CWLS without correction for weak instrument bias. We further applied mtCOJO using the total effects on WHR for comparison with index effect regression.

Second, we consider GWAS for SNPs associated with smoking cessation. Because this can only be conducted among smokers, the analysis is conditional on smoking initiation, and collider bias could plausibly be introduced by common determinants of initiation and cessation. The GSCAN consortium (Liu et al., 2019) identified SNPs in 24 genomic regions associated with smoking cessation (binary current/former smoker) in a total sample of 547,219 individuals. We obtained log odds ratios from that study in the subset of 312,821 individuals excluding the 23andMe sample.

To adjust these associations for collider bias we used summary statistics for smoking initiation from the same study in 632,802 individuals excluding 23andMe. We estimated the regression slope b using 395,941 pruned SNPs ($r^{2}\leq 0.1$, 250 kb window), using the full set and also with *p*  value thresholds of ${5\times 10}^{-8}$, $10^{-5}$, 0.001 and 0.05. We again compared CWLS, MR‐RAPS and Slope‐hunter and additionally applied MR‐Mix. MR‐RAPS was performed within the TwoSampleMR R package (version 0.5.6) and MR‐Mix using the MR‐Mix package (version 0.1.0). For MR analyses, SNPs were pruned using default parameters in the TwoSampleMR package ($r^{2}\leq 0.001$, 10 Mb window). To compare results with those from nonoverlapping samples, we repeated these analyses using only the UK Biobank subjects for smoking initiation (*N * = 461,066) and non‐UK Biobank subjects for smoking cessation (*N * = 143,851).

## 3 RESULTS

### 3.1 Simulations

Properties of the methods have been extensively explored in previous studies (Dudbridge et al., 2019; Mahmoud et al., 2022; Zhu et al., 2018). Here, we report simulations to demonstrate the similarity between MR‐RAPS and CWLS, and to show the effect of positive allele coding in the general MR‐Egger model.

Simulations followed a similar structure to those of Dudbridge et al. (2019) and Mahmoud et al. (2022). We simulated 100,000 independent SNPs under Hardy–Weinberg equilibrium with minor allele frequencies drawn uniformly from (0.01, 0.49). SNP effects, confounders and residual variation in X and Y were drawn independently from normal distributions. 5000 SNPs had effects on X only, 5000 on Y only and 5000 on both X and Y. X and Y were simulated as normally distributed traits with 50% heritability, nongenetic confounder explaining 40% variance and 10% residual variance. Type‐1 error and power for SNP effects on Y conditional on X were estimated at the 5% significance level among the SNPs with effects on X.

Table 2 shows error rates for different genetic correlations between X and Y. The InCLUDE assumption holds only when the genetic correlation is 0. The unadjusted analysis has increased Type‐1 error arising from collider bias, but also has increased power compared to the adjusted results. However, the false discovery rates are generally lower for the adjusted analysis, except under a strong negative correlation. The estimates for MR‐RAPS and CWLS are very similar.

**Table 2:** Error rates compared between methods

Genetic correlation	Method	Type‐1 error	Power	FDR
0	Unadjusted	0.0724	0.202	0.263
	CWLS	0.0505	0.164	0.236
	MR‐RAPS	0.0502	0.164	0.235
	Slope‐hunter	0.0573	0.183	0.238
	MR‐Egger	0.0754	0.205	0.269
0.45	Unadjusted	0.125	0.101	0.553
	CWLS	0.087	0.122	0.416
	MR‐RAPS	0.0872	0.121	0.419
	Slope‐hunter	0.0903	0.121	0.427
	MR‐Egger	0.128	0.0995	0.563
−0.45	Unadjusted	0.0538	0.204	0.209
	CWLS	0.0676	0.0842	0.445
	MR‐RAPS	0.0677	0.0842	0.446
	Slope‐hunter	0.0503	0.165	0.234
	MR‐Egger	0.0556	0.212	0.207

*Note*: Type‐1 error, power (both at *α*  ≤ 0.05) and FDR for tests of SNPs with effects on the conditioning covariate *X*, over 1000 simulations of quantitative *X* and *Y* with parameters given in the main text. FDR was calculated as the ratio of Type‐1 error to the sum of Type‐1 error and power, as there were 5000 SNPs both with and without direct effects on *Y*. Unadjusted, tests based on the conditional effects ${\hat{\beta }}_{GY}^{C}$. MR‐Egger, alleles coded to have positive effects on *X*, that is, ${\hat{\beta }}_{GX}\geq 0$.

Abbreviations: CWLS, corrected weighted least squares; FDR, false discovery rate; MR‐RAPS, Mendelian randomisation using the robust adjusted profile score; SNP, single‐nucleotide polymorphism.

Slope‐hunter performed slightly worse than CWLS and MR‐RAPS under no or positive genetic correlation. This is likely due to the equal numbers of SNPs with and without effects on Y. In more extensive simulations, Slope‐hunter tended to perform better when a majority of SNPs had no effect on Y, in line with its ZEMRA assumption (Mahmoud et al., 2022).

The table also shows increased Type‐1 error rates for CWLS with free intercept (i.e., MR‐Egger), where the alleles are coded to have positive estimated effects on X. This confirms that identifying the positive effect allele in the analysed sample leads to improper correction for weak instrument bias, when very weak IVs are included in the analysis. Although the power is also increased, the false discovery rates are generally worse than for CWLS, the exception being under strong negative correlation.

Table 3 gives summaries of the regression slopes estimated in the simulations. As expected, CWLS and MR‐RAPS are unbiased under no genetic correlation, but exhibit bias when a genetic correlation is present. The two methods gave very similar results: the correlation in estimated slopes was 0.92 under no genetic correlation, 0.98 for correlation 0.45 and 0.88 for correlation −0.45. The mean standard error was also similar, but was underestimated by CWLS, which had a greater empirical standard deviation. This is because we have not allowed for the uncertainty in estimating the weak instrument correction. Thus, MR‐RAPS provides a more efficient estimator of the slope than CWLS, as expected, but CWLS does in fact provide a close approximation to MR‐RAPS. Again these simulations did not favour Slope‐hunter. Its correlation was close to zero with both the other methods in each simulation.

**Table 3:** Summaries of the estimated regression slope in the simulations of Table 2

Genetic correlation	True slope	Method	Mean	Empirical SD	Mean SE
0	−0.4	CWLS	−0.404	0.063	0.040
		MR‐RAPS	−0.400	0.0414	0.0416
		Slope‐hunter	−0.202	0.140	N/A
		MR‐Egger	0.0258	0.004	0.002
0.45	−0.5125	CWLS	−0.178	0.0402	0.0338
		MR‐RAPS	−0.176	0.0338	0.0335
		Slope‐hunter	−0.184	0.146	N/A
		MR‐Egger	0.0116	0.003	0.002
−0.45	−0.2875	CWLS	−0.630	0.0861	0.0421
		MR‐RAPS	−0.624	0.0471	0.048
		Slope‐hunter	−0.175	0.0665	N/A
		MR‐Egger	0.040	0.0036	0.003

*Note*: Genetic correlation, correlation between SNP effects on covariate *X* and outcome *Y*. Mean, mean estimated slope over 1000 simulations. Slope‐hunter computes a bootstrap SE, which we omitted from the simulation owing to time constraints. However, it was observed to be close to the empirical SD in some randomly selected replicates.

Abbreviations: CWLS, corrected weighted least squares; empirical SD, standard deviation of the estimated slope; mean SE, mean of the estimated standard error; MR‐RAPS, Mendelian randomisation using the robust adjusted profile score; N/A, not available; SNP, single‐nucleotide polymorphism.

Finally, we considered how often the direction of effect was changed by adjustment for collider bias. Because estimates close to zero could change sign stochastically, we considered just the SNPs with true effects on the outcome Y and nominally significant associations after adjustment for collider bias. For each method, we estimated the probability that the adjusted effect changes sign to either the correct direction or the incorrect direction. The results in Table 4 suggest that all methods have low rates of changes of direction to significant results. The rates for Slope‐hunter are slightly lower. For zero genetic correlation, the rates of correct and incorrect changes are similar, whereas there are more correct changes with positive correlation and fewer with negative.

**Table 4:** Proportions of SNPs whose effects are nominally significant in the opposite direction after adjustment for collider bias

Genetic correlation	Method	Correct change (X and Y)	Correct change (Y only)	Incorrect change (X and Y)	Incorrect change (Y only)
0	CWLS	0.064	0.051	0.065	0.058
	MR‐RAPS	0.065	0.051	0.067	0.059
	Slope‐hunter	0.028	0.021	0.019	0.017
	MR‐Egger	0.121	0.117	0.371	0.379
0.45	CWLS	0.056	0.040	0.016	0.019
	MR‐RAPS	0.057	0.041	0.016	0.019
	Slope‐hunter	0.035	0.025	0.009	0.010
	MR‐Egger	0.166	0.134	0.335	0.366
−0.45	CWLS	0.031	0.040	0.159	0.117
	MR‐RAPS	0.031	0.040	0.164	0.120
	Slope‐hunter	0.007	0.01	0.029	0.022
	MR‐Egger	0.113	0.117	0.383	0.378

*Note*: Correct (incorrect) change, the adjusted effect is in the same (opposite) direction as the true effect. *X* and *Y*  (*Y*  only), SNP has a true effect on *X*  and *Y*  (*Y* only), leading to the presence (absence) of collider bias.

Abbreviations: CWLS, corrected weighted least squares; MR‐RAPS, Mendelian randomisation using the robust adjusted profile score; SNP, single‐nucleotide polymorphism.

### 3.2 WHR adjusted for BMI

Table 5 shows the slope of the index effect regression estimated by various methods. MR‐RAPS and CWLS give similar results, noting their standard errors, whereas the estimates from Slope‐hunter had a substantially larger magnitude. As expected, standard errors increased as fewer SNPs were included in the regression, and the weak instrument correction had less effect as SNPs were more strongly selected for association with BMI. Perhaps surprisingly, when all SNPs were included the sign of the slope was positive for all methods, although remaining close to zero, except for Slope‐hunter. However, the adjusted effect sizes of the 346 index SNPs were very close to the unadjusted effects (Supporting Information: Table 1), with increased standard errors owing to the estimation of the adjustment. Overall these results suggest collider bias has a minor effect on the primary GWAS findings, as previously suggested (Pulit et al., 2019). Results from Slope‐hunter were somewhat distinct from the other methods, perhaps indicating violation of either the InCLUDE or ZEMRA assumptions. The positive slope estimated by Slope‐hunter when all SNPs are included in the analysis (p≤1) may reflect instability in the clustering algorithm in the presence of many null SNPs for both WHR and BMI. This would support the default procedure of initially thresholding SNPs, but at the cost of increased standard error.

**Table 5:** Regression slopes for WHR adjusted for BMI

	p≤1	$p\leq 10^{-4}$	$p\leq 10^{-6}$	$p\leq 10^{-8}$
IVW	0.00416 (0.00248)	−0.0511 (0.0114)	−0.0807 (0.0169)	−0.0872 (0.0237)
CWLS	0.00848 (0.00506)	−0.053 (0.0118)	−0.0826 (0.0173)	−0.0888 (0.0242)
MR‐RAPS	0.0355 (0.00615)	−0.0353 (0.0129)	−0.0672 (0.0159)	−0.0576 (0.0231)
Slope‐hunter	0.324 (0.0335)	−0.165 (0.0495)	−0.135 (0.070)	−0.217 (0.0447)

*Note*: Estimated slope (s.e.) of the regression of SNP effects on WHR adjusted for BMI on SNP effects on BMI, using SNPs selected by different *p* value thresholds for association with BMI.

Abbreviations: BMI, body mass index; CWLS, corrected weighted least squares; IVW, inverse variance weighted; MR‐RAPS, Mendelian randomisation using the robust adjusted profile score; SNP, single‐nucleotide polymorphism; WHR, waist–hip ratio.

We also calculated adjusted effects for all 143,000 pruned SNPs first using instrument effect regression, adjusting the conditional effects $\beta_{\mathrm{GY}}^{C}$, and then with mtCOJO, adjusting the total effects $\beta_{\mathrm{GY}}^{T}$, which were also available as summary statistics. The adjusted effects from the two methods were very similar for all SNPs, with a correlation of 0.988. The correlation increased to 0.995 among the 681 SNPs associated with BMI at $p<10^{-6}$. These results give empirical support for the analytic equivalence shown in the Methods section.

### 3.3 Smoking cessation

Table 6 shows the estimated slopes from index effect regression by various methods. The estimates are generally similar across methods and *p * value thresholds. Again, standard errors increase with stricter *p * value thresholding, although weak instrument correction has less effect. Adjusted effect sizes for the 24 associated SNPs are given in Supporting Information: Table 2, and again are very similar to the unadjusted effects. Supporting Information: Table 3 shows the estimated slopes from the nonoverlapping sample analysis, showing (for *p* value thresholds less than 1) results consistent with, although less precise than, those of Table 6.

**Table 6:** Regression slopes for smoking cessation

	p≤1	p≤0.05	p≤0.001	$p\leq 10^{-5}$	$p\leq {5\times 10}^{-8}$
IVW	0.180 (0.015)	0.179 (0.016)	0.206 (0.019)	0.258 (0.029)	0.225 (0.033)
CWLS	0.044 (0.004)	0.051 (0.003)	0.114 (0.007)	0.194 (0.014)	0.227 (0.028)
MR‐RAPS	0.198 (0.018)	0.198 (0.018)	0.226 (0.021)	0.287 (0.030)	0.222 (0.033)
Slope‐hunter	−0.604 (0.093)	0.353 (0.024)	0.335 (0.039)	0.487 (0.026)	0.558 (0.065)
MR‐Mix	0.050 (0.200)	0.110 (0.175)	0.120 (0.107)	0.430 (0.249)	2.01 × 10^−17^ (0.705)

*Note*: Estimated slope (s.e.) of the regression of SNP effects on smoking cessation on SNP effects on smoking initiation, using SNPs selected by different *p* value thresholds for association with smoking initiation.

Abbreviations: CWLS, corrected weighted least squares; IVW, inverse variance weighted; MR‐RAPS, Mendelian randomisation using the robust adjusted profile score; SNP, single‐nucleotide polymorphism.

## 4 DISCUSSION

There are strong parallels between the corrections for collider bias described here and the mature field of two‐sample MR. Both approaches aim to estimate a linear relationship between SNP effects on two traits, under IV assumptions on the SNPs. Whereas the same mathematical procedures can be applied to both problems, there are some key differences in context. In the collider bias setting the interest is on the direct effects of SNPs on the outcome trait, with these direct effects expected to exist and being the target of inference. The linear relationship itself is not of primary interest, and we believe that precision is more important than bias in estimating that relationship, to retain the power to detect the direct effects. This contrasts with MR in which unbiased inference of a causal effect is the priority.

For this reason, we advocate methods designed for a larger number of IVs. Here, we have used Slope‐hunter, MR‐RAPS and MR‐Mix, but many other methods are now available (Burgess et al., 2020; Darrous et al., 2021; Morrison et al., 2020). We have described a simple correction to the weighted least‐squares estimator in instrument effect regression, which closely approximates MR‐RAPS when many SNPs are used and provides a useful alternative using elementary methods.

Heckman models have long used IVs to account for selection bias. The contribution of the present methods is the application of IVs to collider bias in linear models, where the same approach can be used for conditioning on a continuous trait and for selection on a binary trait, assuming small effects of IVs. In turn, this allows two‐sample analyses with summary statistics and the use of large numbers of SNPs, which may individually violate the IV assumptions while meeting collective assumptions such as InCLUDE.

The link with MR is explicit in the mtCOJO approach, which subtracts an estimate of the indirect effect from the total association of an SNP with the outcome. In our example of WHR adjusted for BMI, we have shown that this gives equivalent results to instrument effect regression, and both approaches are appropriate for conditioning on a continuous trait, depending on which summary statistics are available. Indeed, both approaches estimate the direct effect of G by subtracting its effect on X, multiplied by the IV‐estimated effect, from an uncorrected effect on Y. Operationally, the same software could be used for either approach, using as the input either the total effects $\beta_{\mathrm{GY}}^{T}$ (for mtCOJO) or conditional effects $\beta_{\mathrm{GY}}^{C}$ (for instrument effect regression), along with the effects on exposure $\beta_{\mathrm{GX}}$.

We have presented results for Type‐1 error, power and false discovery rate, reflecting the emphasis on testing over estimation in GWAS. Parameter estimates are nevertheless important for subsequent analyses, including polygenic scoring and MR and are the direct subject of collider bias. Because the bias varies with the $\beta_{\mathrm{GX}}$ effects, the net bias in a GWAS can be close to zero and it is difficult to characterise a typical bias on individual SNPs. For this reason, we have focussed on error rates as a proxy for estimation bias. In our earlier work, we showed that Type‐1 errors at the nominal level correspond to low absolute bias on parameter estimates (Dudbridge et al., 2019; Mahmoud et al., 2022).

In our data examples, the primary results were barely changed by adjustment for collider bias. This is reassuring but should not be taken to mean that adjustment is unnecessary. Estimating the adjustment, even if small, increases the standard error of the estimates and this may play into subsequent analyses.

In the situations we have considered, SNP associations on the conditioning trait are readily available, as are the associations with the outcome. However, there are other scenarios in which collider biases are not so amenable to the present methods. In case/control GWAS using prevalent cases, the analysis is conditional on individuals surviving until study recruitment and genotyping, with collider bias arising if there are common determinants of short‐term survival and longer‐term disease. This may be a particular issue for acute events such as myocardial infarction (Hu et al., 2017). Correction for such bias would require GWAS of survival to recruitment, but such a study would be difficult to carry out. A similar situation occurs when the process of being tested for disease depends on factors common to the disease itself (Griffith et al., 2020).

Representativeness is a common concern in epidemiological studies, but determinants of study participation may give rise to collider bias (Yaghootkar et al., 2017). GWAS of participation cannot be directly performed, although insight might be gained by comparing genotype frequencies between volunteer‐based studies and birth cohorts, or through linkage of large‐scale studies. MR and mediation analyses, which include additional exposures in the model structure, introduce further scope for collider biases that may be amenable to genetic IVs (Griffith et al., 2020; Munafo et al., 2018; Paternoster et al., 2017).

Currently, therefore, the approaches we have described have limitations. Nevertheless, when there may be unknown confounding between a conditioning trait and an outcome of interest, they do offer a useful addition to the existing toolkit of sensitivity analysis, inverse probability weighting and other model‐based corrections. Indeed genetic IV methods may be applied in conjunction with those approaches to provide improved assessment of and adjustment for collider bias. We expect further developments of genetic IV approaches to address collider bias in applications such as secondary analyses of case/control studies and MR of factors affecting disease progression.

## Supporting information

Supporting information.

Supporting information.

Supporting information.

## Data Availability

The data that support the findings of this study are available from the following resources available in the public domain: GIANT consortium data files, https://portals.broadinstitute.org/collaboration/giant/index.php/GIANT_consortium_data_files_GSCAN_consortium_data_files and https://conservancy.umn.edu/handle/11299/201564.
