# Supplementary material for: Adjusting for collider bias in genetic association studies using instrumental variable methods
Source: Genet Epidemiol. 2022 May 18;46(5-6):303–16. doi: 10.1002/gepi.22455 (PMC9544531; doi:10.1002/gepi.22455)
Supplement: Supplementary file 3 — Supporting information. [file GEPI-46-303-s003.docx]

|  | $P\leq1$ | $P\leq0.05$ | $P\leq0.001$ | $P\leq{10}^{-5}$ | $P\leq{5\times10}^{-8}$ |
| --- | --- | --- | --- | --- | --- |
| IVW | 0.780 (0.072) | 0.159 (0.055) | 0.194 (0.068) | 0.447 (0.103) | 0.524 (0.181) |
| MR-RAPS | 0.981 (0.096) | 0.177 (0.062) | 0.220 (0.074) | 0.497 (0.112) | 0.549 (0.198) |
| MR-Mix | 0.380 (1.910) | 0.390 (0.270) | 0.400 (0.146) | 0.800 (0.838) | 2x10^-17^ (0.623) |

Supplementary table 3. Estimated slope (s.e.) of the regression of SNP effects on smoking cessation on SNP effects on smoking initiation, using SNPs selected by different P-value thresholds for association with smoking initiation. Analysis restricted to UK Biobank subjects for smoking initiation, and non-UK Biobank subjects for smoking cessation.
